# DeepOMe: A Web Server for the Prediction of 2′-O-Me Sites Based on the Hybrid CNN and BLSTM Architecture

**DOI:** 10.3389/fcell.2021.686894

**Published:** 2021-05-14

**Authors:** Hongyu Li, Li Chen, Zaoli Huang, Xiaotong Luo, Huiqin Li, Jian Ren, Yubin Xie

**Affiliations:** ^1^School of Life Sciences, Precision Medicine Institute, The First Affiliated Hospital, Sun Yat-sen University, Guangzhou, China; ^2^School of Computer Science and Engineering, Sun Yat-sen University, Guangzhou, China

**Keywords:** CNN, BLSTM, web service, RNA modification, 2′-O-methylation

## Abstract

2′-O-methylations (2′-O-Me or Nm) are one of the most important layers of regulatory control over gene expression. With increasing attentions focused on the characteristics, mechanisms and influences of 2′-O-Me, a revolutionary technique termed Nm-seq were established, allowing the identification of precise 2′-O-Me sites in RNA sequences with high sensitivity. However, as the costs and complexities involved with this new method, the large-scale detection and in-depth study of 2′-O-Me is still largely limited. Therefore, the development of a novel computational method to identify 2′-O-Me sites with adequate reliability is urgently needed at the current stage. To address the above issue, we proposed a hybrid deep-learning algorithm named DeepOMe that combined Convolutional Neural Networks (CNN) and Bidirectional Long Short-term Memory (BLSTM) to accurately predict 2′-O-Me sites in human transcriptome. Validating under 4-, 6-, 8-, and 10-fold cross-validation, we confirmed that our proposed model achieved a high performance (AUC close to 0.998 and AUPR close to 0.880). When testing in the independent data set, DeepOMe was substantially superior to NmSEER V2.0. To facilitate the usage of DeepOMe, a user-friendly web-server was constructed, which can be freely accessed at http://deepome.renlab.org.

## Introduction

To date, hundreds of different RNA modifications have been identified in human transcriptome, and found to be critical in the regulation of various transcriptional events (Behm-Ansmant et al., 2011). Among those, 2′-O-methylation (2′-O-Me) is one of the most abundant RNA modifications, presenting in transfer RNAs (tRNAs) (Somme et al., 2014), ribosomal RNAs (rRNAs) (Rebane and Metspalu, 2002), small nuclear/small nucleolar RNAs (snRNAs/snoRNAs) (Darzacq et al., 2002), microRNAs (miRNAs) (Li, 2005)/Piwi-interacting RNAs (piRNAs) (Yu et al., 2005), and some messenger RNAs (mRNAs) (Dai et al., 2017). The addition of methyl groups on the ribose moiety can affect sterical properties, hydrogen-bonding potential, and structural rigidity of the target RNA (Kierzek et al., 2009; Hengesbach and Schwalbe, 2014), and orchestrating the biogenesis (Ojha et al., 2020), metabolism (Salem et al., 2019), and functions (Choi et al., 2018) of these RNA molecules. Given its functional importance, the precise detection and functional analysis of 2′-O-Me are important research topics in the community.

Recently, several experimental techniques were developed to pinpoint the precise 2′-O-Me sites. For example, perchloric acid (HClO4) hydrolysis (Baskin and Dekker, 1967), periodate oxidation hydrolysis (Trim and Parker, 1972), chromatography and mass-spectrometry (Abbate and Rottman, 1972; Sardana and Fuke, 1980; Fenghe and Mccloskey, 1999; Kirpekar et al., 2005). At present, high-throughput techniques that established based on deep sequencing were also reported. Typical examples included RiboMethSeq (Krogh et al., 2016; Erales et al., 2017; Sharma et al., 2017; Zhou et al., 2017), 2′-OMe-Seq (Incarnato et al., 2017), RibOxi-Seq (Zhu et al., 2017), and Nm-seq (Dai et al., 2017; Hsu et al., 2019).

Although the previous mentioned high-throughput techniques can provide single-nucleotide mapping of 2′-O-Me sites at transcriptome level, the experimental procedure is still expensive and labor-exhausting. Therefore, there is still an urgent need of a computational model to mine the sequence feature of 2′-O-Me sites and identify the 2′-O-Me sites *in silico*. So far, several computational methods such as iRNA-2methyl (Qiu et al., 2017), Deep-2′-O-Me (Mostavi et al., 2018), iRNA-2OM (Yang et al., 2018), NMSEER V2.0 (Zhou et al., 2019), and iRNA-PseKNC (Tahir et al., 2019) have been developed. However, many issues remain in these methods, leaving plenty of room for improvement. Firstly, 2′-O-Me can occur in all types of RNA nucleotides, resulting an extremely imbalanced dataset between positive and negative samples. The traditional classification algorithm, which aims at the overall classification accuracy, pays too much attention to the major class, leading to poor performances in minor class and high false positives. Secondly, previous studies have randomly sampled subsequences near experimentally identified 2′-O-Me sites as negative sequences. This procedure can produce a high degree of similarity between extracted positive and negative sequences in training dataset, which limits the accuracy of traditional sequence-based models. Third, many tools lack a convenient webserver, hindering their widespread use in biological scenario. Therefore, the development of a reliable prediction tool that can not only extract useful features from the primary mRNA sequences but also produce high-precision results is still an important problem to be solved.

The performance of traditional machine learning algorithms relies heavily on data representations. However, features are typically designed by human engineers with extensive domain expertise, and identifying which features are more appropriate for the given task remains difficult. Thanks to the ability of deep learning architectures in automatically extracting high-representation information in the raw data, the application of deep learning framework is a promising way to address the above issues. In recent years, many attempts have been made to apply deep learning algorithms in biological research. For example, DeepBind (Alipanahi et al., 2015) for predicting DNA- and RNA-binding specificity, AlphaFold (Senior et al., 2020) for predicting protein structure, scDeepCluster (Tian et al., 2019) for clustering single cell RNA-seq data, DeepCpG (Angermueller et al., 2017) for predicting single-cell DNA methylation state. Considering the characteristics of 2′-O-Me, deep learning algorithms are more suitable to analyses the patterns of 2′-O-Me and thus may greatly improve the prediction performance.

In this article, we present DeepOMe, a web server based on a hybrid deep learning architecture for predicting 2′-O-Me sites in Human mRNA. To our best knowledge, our work is the first effort to use the combination of CNN with RNN in the prediction of mRNA modification sites under the sequence-to-sequence mode. Moreover, a webserver was further developed and makes it easier for researchers and experimenters to use our proposed model.

## Materials and Methods

### Dataset Collection

The training and test dataset of DeepOMe was constructed from the recently developed Nm-seq experiment (Dai et al., 2017) which comprised of 4,481 2′-O-Me sites in human transcriptome. The site data were first preprocessed and split into training and independent test set using the scheme presented in Supplementary Figure 1. Firstly, 2′-O-Me sites in intergenic region were removed. Due to the reason that our collected data had two coordinates versions (GRCh37 and GRCh38), we next converted original GRCh37 coordinates to GRCh38 coordinates using LiftOver and further mapped it to human transcripts for better transcriptome annotation. Transcript sequences with at least one mapped 2′-O-Me site were extracted according to the corresponding gene set annotation. If the same 2′-O-Me site located in multiple transcripts, the longest transcript were retained in our dataset. Finally, we collected 2,285 RNA sequences with 3,052 2′-O-Me sites as the final data set. We randomly selected 10% of the collected RNA sequences as independent testing set, and the remaining sequences were regarded as training set. As the result, we assembled 2,046 sequences with 2,743 2′-O-Me sites as the training set, and 239 sequences with 309 2′-O-Me sites as the testing set.

### Extract Features From Primary mRNA Sequences

As mentioned above, previous studies extracted the flanking region of specific length around each 2′-O-Me site as the positive sequences for the training process. To create non-2′-O-Me sites or negative sequences, they randomly selected the non-modified RNA sites around known 2′-O-Me sites and captured its surrounding nucleotide sequences as negative sequences. This procedure suffers from several pitfalls.

First of all, the training set would contain overlapping sequences if the randomly selected negative sites were adjacent to 2′-O-Me sites. This would result in high similarity between positive sequences and negative sequence. The similar sequences would generate many redundant sequence-based features and thus make sequence-based machine learning algorithms difficult to train a validity predictor. To avoid such a scenario, a positive-to-negative ratio (1:10 in NmSEER V2.0; 1:1 in iRNA-2OM; 1:4 in Deep-2′-O-Me) in training set should be manually set. Since in natural transcripts the number of 2′-O-Me sites and non-2′-O-Me sites are highly imbalanced, this kind of operations may always generate many false positives.

**FIGURE 1:**
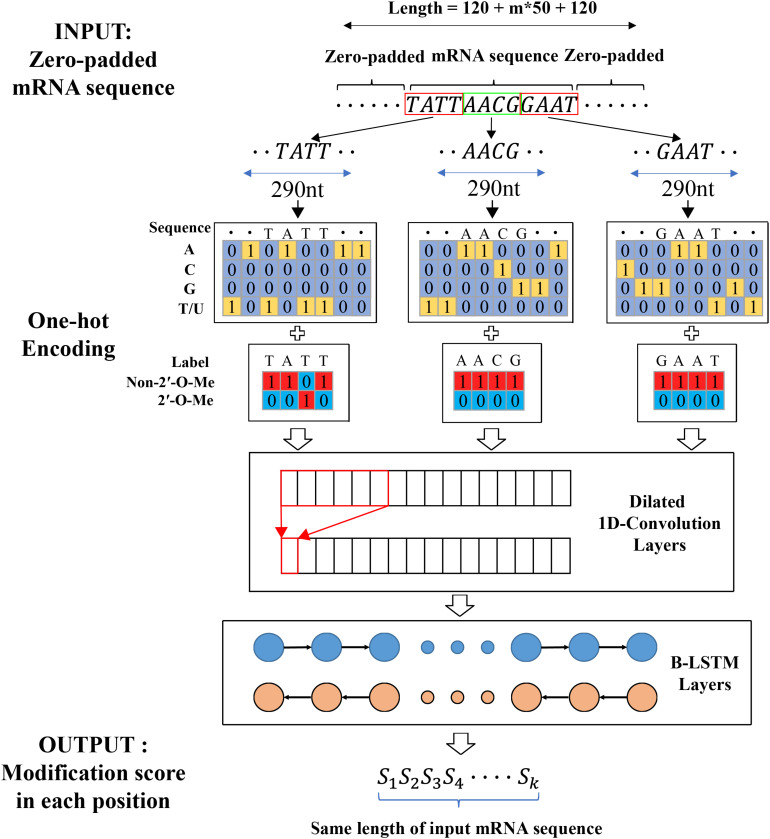
The workflow of predicting 2′-O-Me sites from primary mRNA sequences. For splitting the input mRNA sequence into blocks, DeepOMe uses flanking sequence length of 120 and then predicts whether each position in extracted blocks contains 2′-O-methylation.

To solve these problems, the similar procedure from Jaganathan et al. (2019) was chosen to extract input features and output labels from primary mRNA sequences (as shown in Figure 1). Firstly, the transcript sequence was one-hot encoded as follows: A, C, G, T/U mapped to [1,0,0,0], [0,1,0,0], [0,0,1,0], [0,0,0,1], respectively. Next, the one-hot encoded sequence was zero-padded until the length became a multiple of 50 in order to successfully split into non-overlapped blocks of 50 nt. To capture sequence dependent features, such mRNA sequence was further zero-padded at the 5′- and 3′-end with a flanking sequence of length *L*. At last, the padded sequence was split into blocks in such a way that the *i*^*th*^ block consisted of nucleotide positions from 50(*i*−1)−*L* + 1 to 50*i* + *L*. Therefore, the 50nt center regions in the *i*^*th*^ block and (*i* + 1)*^*th*^* block had no overlapping sequence in original mRNA sequence. Similarly, the modification output label sequence was one-hot encoded as follows: 2′-O-Me modification and non-2′-O-Me modification were mapped to [0,1] and [1,0] respectively. The one-hot encoded label sequence was zero-padded until the length became a multiple of 50 and then further zero-padded at the start and the end with a flanking sequence of length *L*. The padded label sequence was split into blocks using the same procedure as described for the inputted mRNA sequence. The extracted one-hot encoded nucleotides sequences and the corresponding one-hot encoded label sequences were used as inputs and the target outputs to train and evaluate our model.

### Architecture of the CNN-BLSTM Model

**FIGURE 2:**
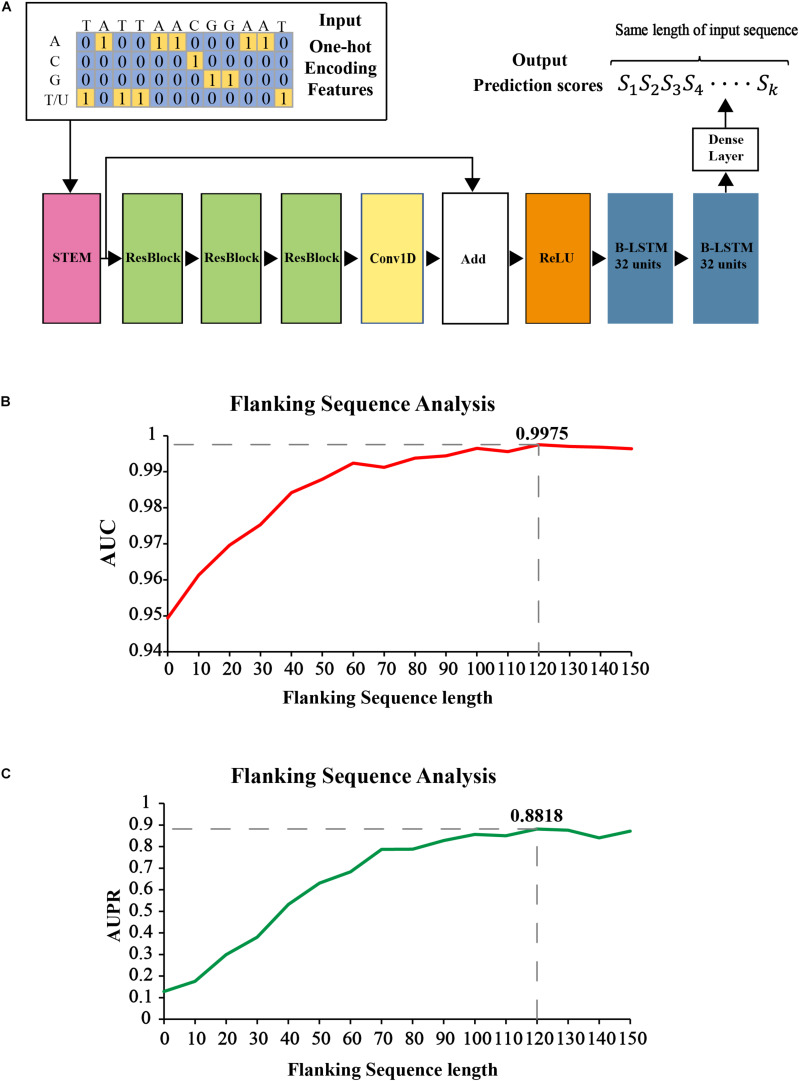
The construction of prediction model in DeepOMe. **(A)** Network architecture of the DeepOMe prediction model. Flanking sequence selection under 4-fold cross-validation by AUPR **(B)** and AUROC **(C)**.

Figure 2A shows our proposed CNN-BLSTM architecture. DeepOMe is composed of 10 layers of CNN and 2 layers of B-LSTM (Schuster and Paliwal, 1997). The model structure consists of input layer, CNN layers, BLSTM layers, fully connected layer, and the output layer. The input layer can receive one-hot encoded sequence data. In the CNN layers, we first enriched the representation in the Stem Block (Supplementary Figure 2A) by computing multiple feature maps with different kernel sizes (Szegedy et al., 2015). Then, we stacked three residual blocks (He et al., 2016) for local feature extraction. The convolution operation in Stem block and ResBlock was 1D-convolution with kernel size of 10 and dilation rate of 2 (Supplementary Figure 2B). The CNN layers were used as preprocessing step to extract the deep spatial features from the input sequences. Then, these deep features were fed into two BLSTM layers with 32 units for learning of sequence-dependent features. The last layer in our model is a fully-connected layer with softmax activation, which was used to generate the final prediction score. The detailed architectural information was listed in Supplementary Table 1.

### Experimental Setup

The proposed model was implemented with the TensorFlow library (Abadi et al., 2016) in Python and trained on an NVIDIA GTX2080 GPU. The proposed model was trained through 100 epochs using batch size of 200. The categorical cross entropy loss between the target and the predicted outputs was minimized using Adam optimizer (Kingma and Ba, 2014). The initial learning rate of 0.001 was used to train the model. Early-stopping (Caruana et al., 2001) was used to control overfitting. We monitored the validation loss at each epoch. When the validation loss has not improved after ten epochs, training is interrupted.

### Evaluation Metrics

Testing set was used to validate our proposed model comparing with available prediction tools after cross-validation. The performance was evaluated based on several metrics, namely area under Precision-Recall Curve (AUPR), area under Receiver Operating Characteristic Curve (AUC), sensitivity (Sn), specificity (Sp), precision (Pr), accuracy (Acc), and Matthew’s correlation coefficient (Mcc).

When evaluating model’s performance in full mRNA sequence, an accuracy metric was largely ineffective since most of the positions in mRNA sequence are not 2′-O-Me sites. The prediction model was like the recommender systems which was to suggest the most proper modification sites in mRNA sequences. Thus, top-k accuracy was more appropriate in such situation. When comparing among different methods, we evaluated the top-k accuracy besides the AUC and AUPR metrics. The top-k accuracy is defined as follows: Suppose the test set has k positions that belong to the right class which is 2′-O-Me site. We choose the threshold so that exactly k test set positions are predicted as belonging to the right class. The fraction of these k predicted positions that truly belong to the right class is reported as the top-k accuracy.

## Results

### Flanking Sequence Length Analysis

It is necessary to determine the optimal flanking sequence length *L* of input sequences for identifying 2′-O-Me sites. Generally speaking, if the flanking sequence around the known 2′-O-Me site is too short, it may not carry enough information for prediction and will lead to poor performance. Otherwise, If the flanking sequence is too long, it may carry too much redundant information, leading to poor generalization. Thus, we first analyzed the averaged AUC and AUPR of the proposed model with different flanking sequence length under 4-fold cross-validation. As shown in Figures 2B,C the search step size for flanking sequence length was 10 nt, with a range of 0 and 150. According to the evaluation results, when the flanking sequence length equals to 120 nt and block length equals to 290 nt, the performance generated by our proposed model was the best (AUC = 0.9975, AUPR = 0.8818). Therefore, we selected the flanking sequence with length of 120.

### Evaluation of the Prediction Performance

**FIGURE 3:**
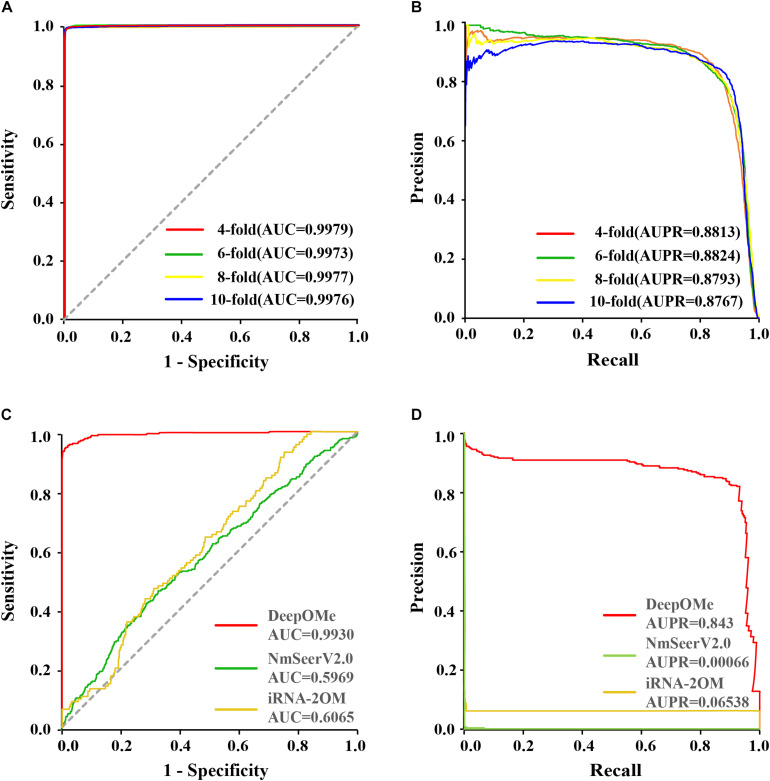
Performance evaluation and comparison. The ROC **(A)** and PR **(B)** curves in 4-, 6-, 8-,10-fold cross-validation. The ROC **(C)** and PR **(D)** curves in testing set between DeepOMe, NmSEER V2.0 and iRNA-2OM.

To evaluate the prediction performance of DeepOMe, we performed 4-, 6-, 8-, and 10-fold cross-validation of the training set. Figures 3A,B shows the ROC and PR curves of our proposed CNN-BLSTM model under 4-, 6-, 8-, and 10-fold cross-validations with flanking sequence length of 120. As a result, DeepOMe showed an acceptable performance in n-fold cross-validations with the area under the ROC curves (AUROC) close to 0.998 and area under the PR curves (AUPR) close to 0.880.

To rigorously evaluate the prediction and generalizability performance of DeepOMe, we next compared it with other state-of-art predictors using the independent test set. Since only iRNA-2OM, iRNA-2methyl, and NMSEER V2.0 provided web-server or standalone package for usage, the comparison will only perform between them. During the comparison, we further found that there were no responses in the webservers of iRNA-2methyl, hence, the final comparison only performs between DeepOMe, NmSEER V2.0, and iRNA-2OM.

Figures 3C,D presented the comparison results in ROC curves and PR curves. The results showed that the DeepOMe achieved a better performance (AUROC = 0.993, 95%CI:0.993-0.993; AUPR = 0.843) in the testing set than NmSEER V2.0(AUROC = 0.5969, 95%CI:0.599-0.600; AUPR = 0.00066) and iRNA-2OM (AUROC = 0.6065, 95%CI:0.601–0.612; AUPR = 0.06538). When testing in full mRNA sequences, we further compared the top-k accuracy between DeepOMe, NmSEER V2.0, and iRNA-2OM. The comparison results in Table 1 suggested that DeepOMe (Top-1 Acc = 0.8602, Top-100 Acc = 0.9563) was more sensitive and robust than NmSEER V2.0(Top-1 Acc = 0.0, Top-100 Acc = 0.1004) and iRNA-2OM (Top-1 Acc = 0.0, Top-100 Acc = 0.1087).

**TABLE 1:** Comparison of Top-k Accuracy between DeepOMe, NmSEER V2.0, and iRNA-2OM in testing set.

Top-k Accuracy	DeepOMe	NmSEER V2.0	iRNA-2OM
Top-1 Accuracy	0.8602	0.0	0.0
Top-3 Accuracy	0.9039	0.0131	0.0
Top-5 Accuracy	0.9082	0.0175	0.0
Top-10 Accuracy	0.9126	0.0175	0.0043
Top-20 Accuracy	0.9257	0.0306	0.0130
Top-30 Accuracy	0.9344	0.0611	0.0130
Top-40 Accuracy	0.9476	0.0699	0.0261
Top-50 Accuracy	0.9520	0.0699	0.0478
Top-60 Accuracy	0.9520	0.0830	0.0696
Top-70 Accuracy	0.9520	0.0917	0.0739
Top-80 Accuracy	0.9563	0.0961	0.0826
Top-90 Accuracy	0.9563	0.1004	0.0870
Top-100 Accuracy	0.9563	0.1004	0.1087

To evaluate the sequence similarity between the predicted sites and the detected sites in transcriptome, sequence logos were generated using WebLogo (Crooks et al., 2004) in training and testing set. Supplementary Figure 3 presented the graphical representation of sequence similarity. The results showed that the predicted sites under different thresholds were similar to the detected sites both in training set and testing set, proving that our proposed model could precisely identity 2′-O-Me sites.

### Web-Server

**FIGURE 4:**
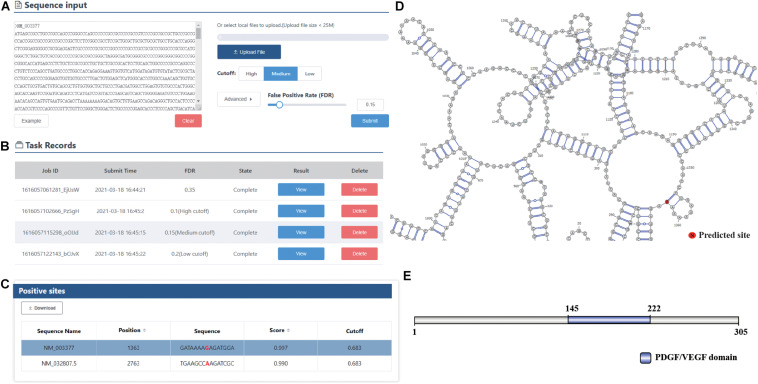
**(A)** The main interface of DeepOMe. mRNA sequences can be input into the text area or uploaded as a single FASTA file. Thresholds with high, medium, and low stringencies are provided in the options panel. **(B)** The submitted task can be checked in the interactive table. **(C)** The result page of DeepOMe. Detailed information for the predicted modification sites, such as modified position, flanking sequence, prediction score, and prediction threshold, is listed in the table. **(D,E)** The visualization results of RNA secondary structure and protein domain organization for the input mRNA sequence using ViennaRNA, IBS, and InterProScan.

To facilitate the use of our prediction models, we next developed an online predictor called DeepOMe for the community. The predictor is freely available at http://deepome.renlab.org. DeepOMe only requires mRNA sequences to run a prediction. Multiple mRNA sequences can be input into the text area or uploaded as s single FASTA file. For users’ convenience, we selected three thresholds based on the 10-fold cross-validation results (Figure 4A), which correspond to the false discovery rate of 0.10, 0.15, and 0.20. The detailed performance values under these three thresholds are shown in Supplementary Table 2. Besides, users can select the threshold by setting the false discovery rate in advanced option menu. After the query sequences are submitted to DeepOMe, users can check its running status in the result panel in real time. When the prediction is complete, the button that links out to the result page will be clickable (Figure 4B). Figure 4C provides a snapshot for the result page of the example mRNA sequence. The prediction position, score and prediction threshold were listed in an interactive table, which allows the users to easily search and sort the results. Remarkably, to facilitate a further analysis of the protein function and RNA structure, we also implemented an automatic pipeline for visualizing the prediction results. By integrating IBS (Liu et al., 2015), InterProScan (Jones et al., 2014), and ViennaRNA (Lorenz et al., 2011) into the web server, DeepOMe can present the graphical representation of the input mRNA sequence together with their predicted sites in the visualization panel. Figures 4D,E provide snapshots for the visualization results of RNA secondary structure and protein domain organization. The diagrams can be saved as a vector graphic (SVG) for further analysis.

## Discussion

2′-O-methylation plays critical roles in regulating gene expressions at the post-transcriptional levels. Thus, proper identification of the 2′-O-Me site is essential to understand the mechanism of RNA metabolisms. 2′-O-Me can occur in any base on the mRNA sequence. Given a mRNA sequence, we need to get an output sequence with the same length of input sequence. The score in each position of output sequence represents whether this position in the input mRNA contains 2′-O-Me. Therefore, the 2′-O-Me site prediction problem can be considered as a many-to-many prediction problem.

However, previous studies tried to train the prediction model based on a Many-to-One mode. They had to randomly select non-2′-O-Me sites around the known 2′-O-Me sites as negative samples, which resulted in high sequence similarity between positive and negative sequences. Besides, to reduce the degree of imbalance in their training data set, the negative sites were manually down-sampled to obtain a relatively small positive-to-negative ratio. However, in reality, the positive-to-negative ratio in a given RNA sequence was always extremely high, and thus caused their models to have poor generalization ability in unseen data. These were the two main reasons why their models received very poor performance in our testing set.

Unlike the previous works that use handcrafted features for classification, DeepOMe could automatically extract the deep features from primary mRNA sequences by CNN layers. DeepOMe was proven to be more efficient than the available method in terms of all evaluation metrics. We found several factors that may explain the high performance achieved by our proposed model. Firstly, the procedure we used to train and test the models was the many-to-many mode. Thus, there was no need to manually balance the training dataset in our model, allowing to learn sufficient information between 2′-O-Me and non-2′-O-Me sites and achieving lower false positives. Secondly, the use of the dilated 1D CNN compared to the traditional 1D CNN allowed our model to cover more relevant information by increasing the receptive filed of the filters. Additionally, the stacked Resblocks used in our model allowed us to build a deeper network and take advantages of the powerful representational ability of deep neural network. At last, the application of bidirectional LSTM was able to exploit meaningful representations from upstream and downstream sequences. The comparison results suggest that the combination of CNN and BLSTM can successfully capture the key features of the entire mRNA sequences. Although promising performance was obtained in DeepOMe, a number of future improvements are expected. First of all, we have designed only a relatively simple CNN-based neural network model in our current version. Various deeper and wider CNN architectures were awaited exploration in the future. Secondly, attention mechanism will be introduced to achieve better representation for contextual information in the future version. Last but not least, we have now trained the model only based on the experimental 2′-O-Me data for *Homo sapiens*. The prediction models for other species such as *Mus musculus* will be established in the future.

## Data Availability Statement

The original contributions presented in the study are included in the article/Supplementary Material, further inquiries can be directed to the corresponding authors.

## Author Contributions

HL implemented the DeepOMe algorithm and wrote the manuscript. LC and XL manually collected 2′-O-Me data from published literatures and performed data pre-processing. ZH and HL are respectively responsible for the front-end page display and back-end logic design of the DeepOMe website. JR was responsible for supervision, funding acquisition, and writing-review. YX supervised this work, reviewed and edited the manuscript. All authors have read and approved the manuscript.

## Conflict of Interest

The authors declare that the research was conducted in the absence of any commercial or financial relationships that could be construed as a potential conflict of interest.
