# Supplementary material for: DeepOMe: A Web Server for the Prediction of 2′-O-Me Sites Based on the Hybrid CNN and BLSTM Architecture
Source: Front Cell Dev Biol. 2021 May 14;9:686894. doi: 10.3389/fcell.2021.686894 (PMC8160107; doi:10.3389/fcell.2021.686894)

DeepOMe: a web server for the prediction of 2′-O-Me sites based on the hybrid CNN and BLSTM architecture

Supplementary Material

# Supplementary Tables

**Supplementary Table 1.** The detailed architectural information for proposed model.

**Supplementary Table 2.** Prediction performance for 2′-O-Me modification with different thresholds.

# Supplementary Figures

**Supplementary Figure 1.** The flowchart of data collection and preprocessing.

**Supplementary Figure 2.** (A) The detailed architecture for Stem Block. (B) The detailed architecture for Resblock.

**Supplementary Figure 3.** The graphical representation of sequence similarity between detected sites and predicted sites. Ground truth denotes the detected sites in training and testing set. High, Medium, Low denotes the predicted sites in training and testing set, which corresponded to high, medium and low thresholds respectively.

**Supplementary Table 1 - Architectural details of the proposed model**

| Layer (Type) | Hyperparameter Details | Output Shape | Connected to |
| --- | --- | --- | --- |
| Input | ─ | (None,290,4) | ─ |
| conv1d(Conv1D) | kernelsize=1 filters=32 | (None,290,32) | Input |
| conv1d_1(Conv1D) | kernelsize=3 filters=32 | (None,290,32) | Input |
| conv1d_2(Conv1D) | kernelsize=5 filters=32 | (None,290,32) | Input |
| batch_normalization (BatchNorm) | ─ | (None,290,32) | conv1d |
| batch_normalization_1(BatchNorm) | ─ | (None,290,32) | conv1d_1 |
| batch_normalization_2(BatchNorm) | ─ | (None,290,32) | conv1d_2 |
| relu (ReLU) | ─ | (None,290,32) | batch_normalization |
| relu_1(ReLU) | ─ | (None,290,32) | batch_normalization_1 |
| relu_2(ReLU) | ─ | (None,290,32) | batch_normalization_2 |
| concatenate (Concatenate) | ─ | (None,290,96) | relu, relu_1, relu_2 |
| conv1d_3(Conv1D) | kernelsize=10 filters=32 dilation rate = 2 | (None,290,32) | concatenate |
| batch_normalization_3(BatchNorm) | ─ | (None,290,32) | conv1d_3 |
| relu_3(ReLU) | ─ | (None,290,32) | batch_normalization_3 |
| conv1d_4(Conv1D) | kernel size=10 filters=32 dilation rate = 2 | (None,290,32) | relu_3 |
| batch_normalization_4(BatchNorm) | ─ | (None,290,32) | conv1d_4 |
| relu_4(ReLU) | ─ | (None,290,32) | batch_normalization_4 |
| conv1d_5(Conv1D) | kernel size=10 filters=32 dilation rate = 2 | (None,290,32) | relu_4 |
| add (Add) | ─ | (None,290,32) | conv1d_3, conv1d_5 |
| batch_normalization_5(BatchNorm) | ─ | (None,290,32) | add |
| relu_5(ReLU) | ─ | (None,290,32) | batch_normalization_5 |
| conv1d_6(Conv1D) | kernel size=10 filters=32 dilation rate = 2 | (None,290,32) | relu_5 |
| batch_normalization_6(BatchNorm) | ─ | (None,290,32) | conv1d_6 |
| relu_6(ReLU) | ─ | (None,290,32) | batch_normalization_6 |
| conv1d_7(Conv1D) | kernel size=10 filters=32 dilation rate = 2 | (None,290,32) | relu_6 |
| add_1(Add) | ─ | (None,290,32) | add, conv1d_7 |
| batch_normalization_7(BatchNorm) | ─ | (None,290,32) | add_1 |
| relu_7(ReLU) | ─ | (None,290,32) | batch_normalization_7 |
| conv1d_8(Conv1D) | kernel size=10 filters=32 dilation rate = 2 | (None,290,32) | relu_7 |
| batch_normalization_8(BatchNorm) | ─ | (None,290,32) | conv1d_8 |
| relu_8(ReLU) | ─ | (None,290,32) | batch_normalization_8 |
| conv1d_9(Conv1D) | kernel size=10 filters=32 dilation rate = 2 | (None,290,32) | relu_8 |
| add_2(Add) | ─ | (None,290,32) | add_1, conv1d_9 |
| conv1d_10(Conv1D) | kernel size=10 filters=32 dilation rate = 2 | (None,290,32) | add_2 |
| relu_9(ReLU) | ─ | (None,290,32) | conv1d_10 |
| bidirectional (BidirectionalLSTM) | units=32，dropout=0.2 | (None,290,32) | relu_9 |
| bidirectional_1(BidirectionalLSTM) | units=32，dropout=0.2 | (None,290,32) | bidirectional |
| dense (Dense Layer) | activation=’softmax’ | (None,290,2) | bidirectional_1 |
| Output | ─ | (None,290,2) | dense |

**Supplementary Table 2 - Prediction performance with different thresholds**

|  | Cutoff | Precision | Specificity | Sensitivity | Accuracy | MCC |
| --- | --- | --- | --- | --- | --- | --- |
|  | >0.977 | 0.950 | 0.999 | 0.861 | 0.999 | 0.904 |
|  |  |  |  |  |  |  |
| High | >0.822 | 0.900 | 0.999 | 0.972 | 0.999 | 0.929 |
|  |  |  |  |  |  |  |
| Medium | >0.683 | 0.85 | 0.999 | 0.979 | 0.999 | 0.912 |
|  |  |  |  |  |  |  |
| Low | >0.520 | 0.8 | 0.999 | 0.983 | 0.999 | 0.887 |
|  |  |  |  |  |  |  |
|  | >0.371 | 0.75 | 0.999 | 0.989 | 0.999 | 0.861 |
|  |  |  |  |  |  |  |
|  | >0.267 | 0.7 | 0.999 | 0.990 | 0.999 | 0.833 |
|  |  |  |  |  |  |  |
|  | >0.167 | 0.65 | 0.999 | 0.992 | 0.999 | 0.803 |

*Note*: The prediction thresholds were selected from 10-fold cross-validation. Specifically, low, medium, and high thresholds were selected under false discovery rate (1 - Precision) of 0.20, 0.15, and 0.10, respectively. MCC, Matthews correlation coefficient.

**Supplementary Figure 1.** The flowchart of data collection and preprocessing.


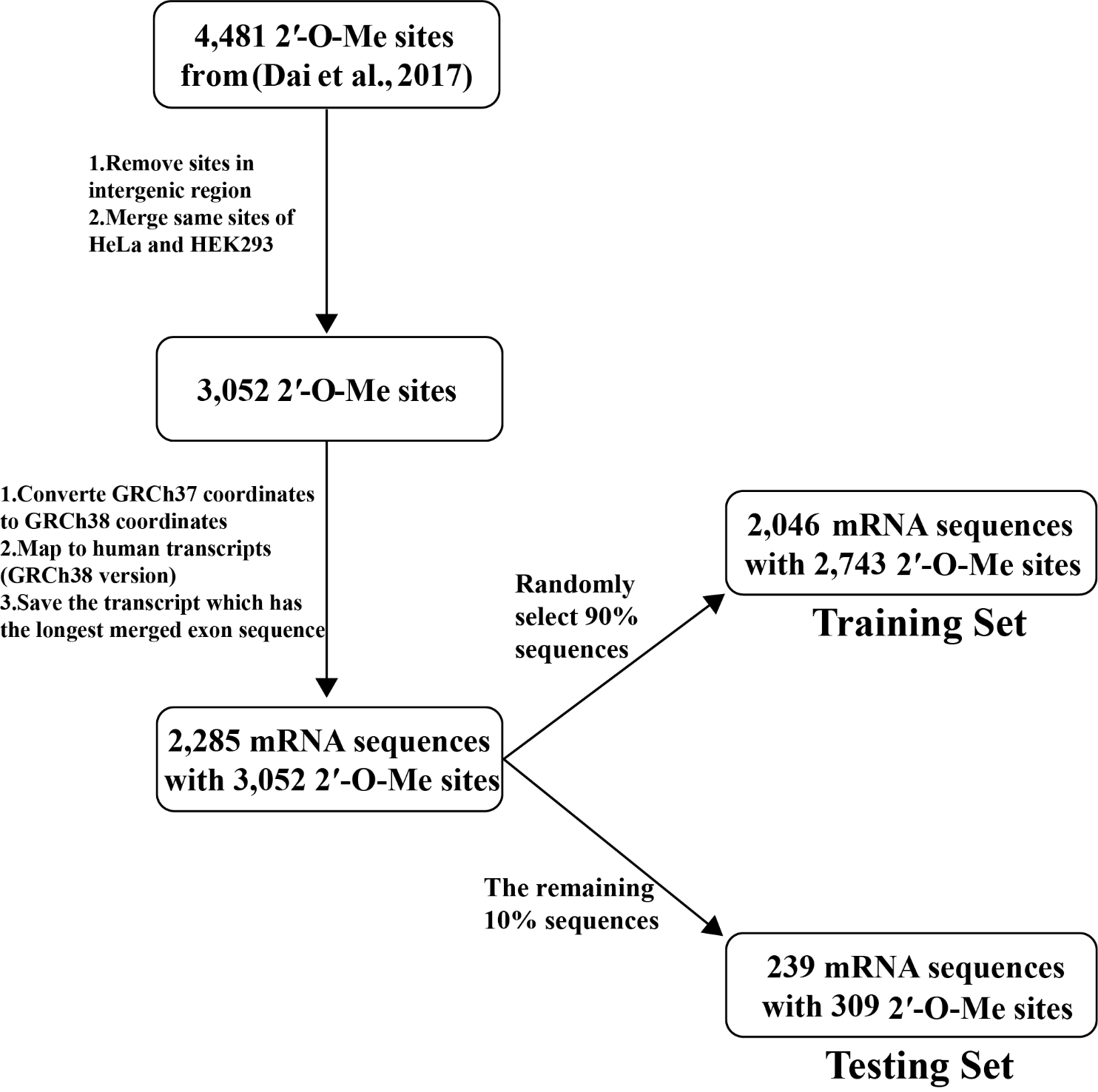


**Supplementary Figure 2.** (A) The detailed architecture for Stem Block. (B) The detailed architecture for Resblock.


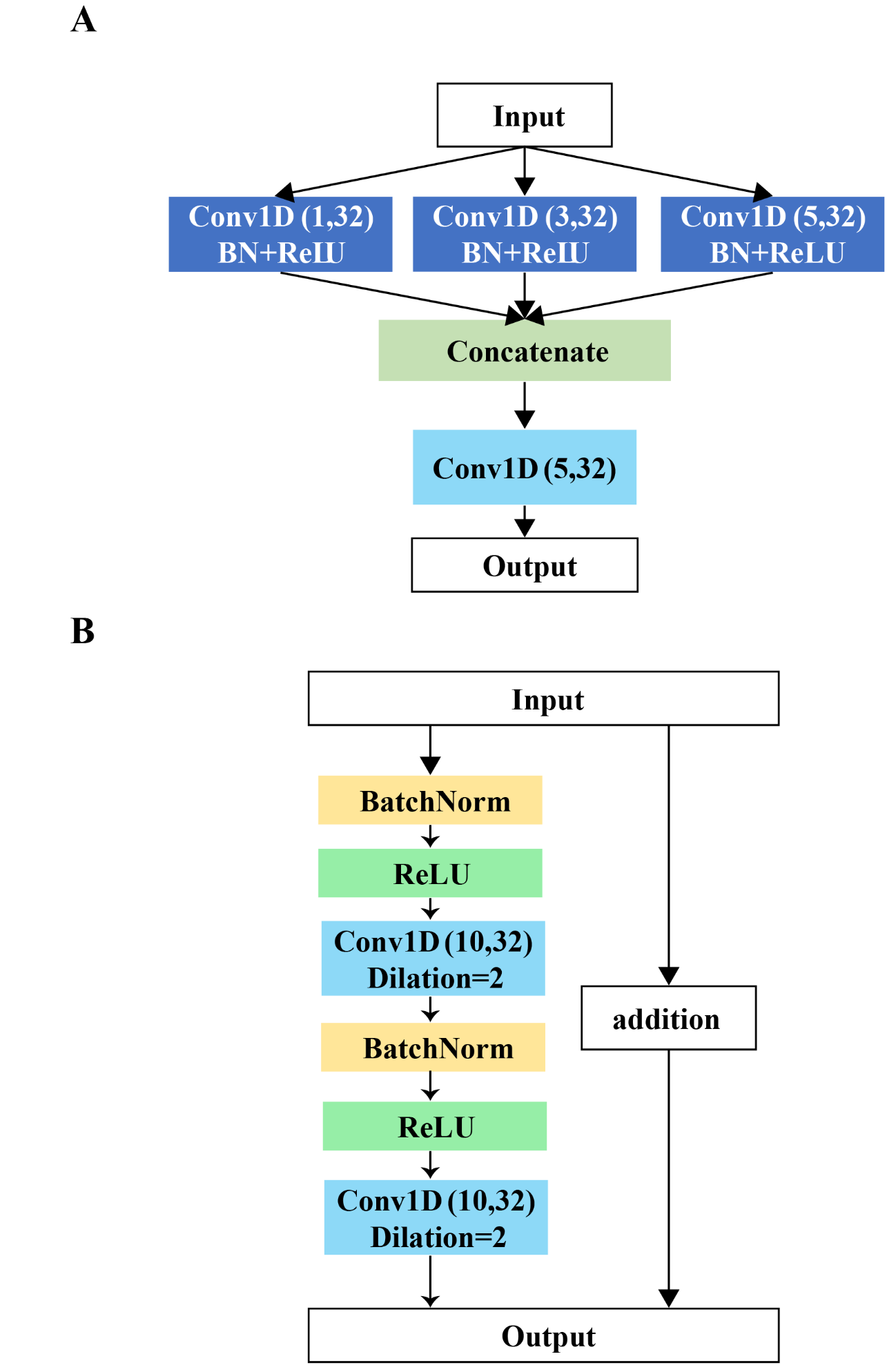


**Supplementary Figure 3.** The graphical representation of sequence similarity between detected sites and predicted sites. Ground truth denotes the detected sites in training and testing set. High, Medium, Low denotes the predicted sites in training and testing set, which corresponded to high, medium and low thresholds respectively.


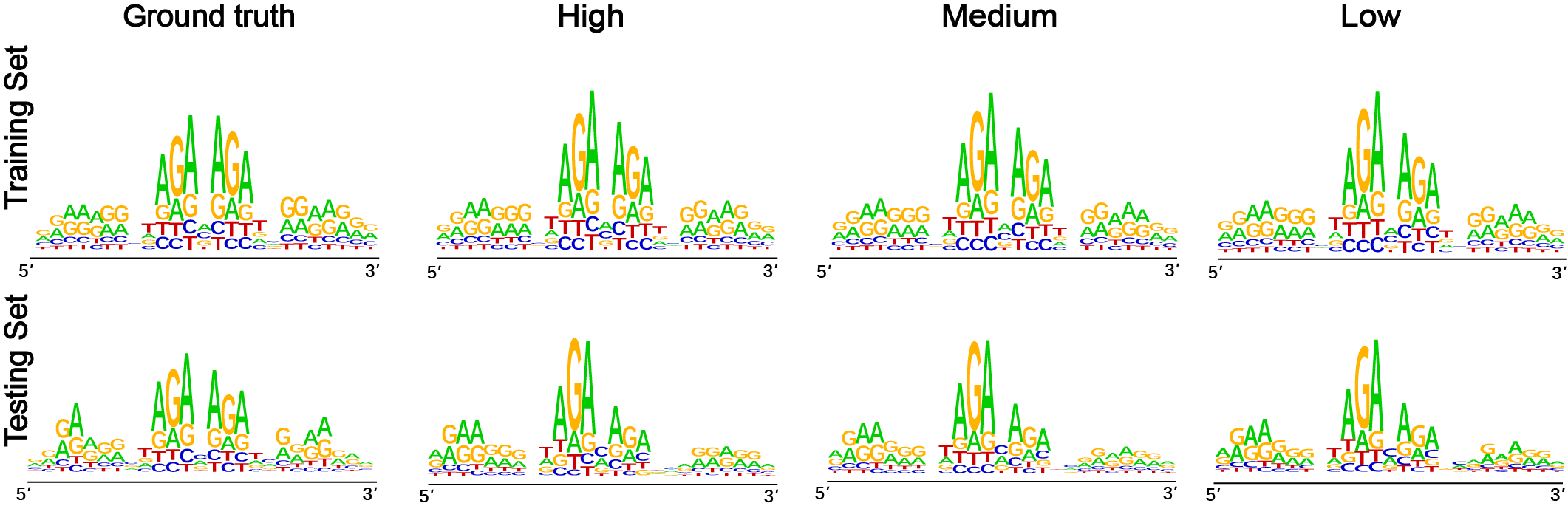

Supplement: Supplementary file 1 [file Data_Sheet_1.docx]
